# High-Affinity Human Anti-c-Met IgG Conjugated to Oxaliplatin as Targeted Chemotherapy for Hepatocellular Carcinoma

**DOI:** 10.3389/fonc.2019.00717

**Published:** 2019-08-02

**Authors:** Yilan Ma, Mingjiong Zhang, Jiayan Wang, Xiaochen Huang, Xingwang Kuai, Xiaojuan Zhu, Yuan Chen, Lizhou Jia, Zhenqing Feng, Qi Tang, Zheng Liu

**Affiliations:** ^1^Medical Center for Digestive Diseases, Second Affiliated Hospital, Nanjing Medical University, Nanjing, China; ^2^Key Laboratory of Antibody Techniques of National Health Commission, Nanjing Medical University, Nanjing, China; ^3^Department of Pathology, Nanjing Medical University, Nanjing, China; ^4^Otorhinolaryngological Department, Second Affiliated Hospital, Nanjing Medical University, Nanjing, China; ^5^Jiangsu Key Lab of Cancer Biomarkers, Prevention and Treatment, Collaborative Innovation Center for Cancer Personalized Medicine, Nanjing Medical University, Nanjing, China

**Keywords:** c-Met, oxaliplatin, antibody-drug conjugate, hepatocellular carcinoma, targeted chemotherapy

## Abstract

Hepatocellular carcinoma (HCC) is one of the most mortality-causing solid cancers globally and the second largest cause of death among malignancies. Oxaliplatin, a platinum-based drug, has been widely utilized in the treatment of malignancies such as colorectal cancer and hepatocellular carcinoma, yet its usage is limited because of severe side effects of cytotoxicity to normal tissues. c-Met, a receptor tyrosine kinase, is expressed aberrantly on the surface of HCC. The purpose of this study was to synthesise a humanized antibody against c-Met (anti-c-Met IgG) and conjugate it to oxaliplatin to develop a novel antibody-drug conjugate (ADC). Anti-c-Met IgG was detected to be loaded with ~4.35 moles oxaliplatin per mole of antibody. ELISA and FCM confirmed that ADC retained a high and selective binding affinity for c-Met protein and c-Met-positive HepG2 cells. *In vitro*, the cytotoxicity tests and biological function assay indicated that ADC showed much higher cytotoxicity and functioning in c-Met-positive HepG2 cells, compared with shMet-HepG2 cells expressing lower levels of c-Met. Furthermore, compared with free oxaliplatin, ADC significantly improved cytotoxicity to c-Met-positive tumours and avoided off-target cell toxicity *in vivo*. In conclusion, by targeting c-Met-expressing hepatoma cells, ADC can provide a platform to reduce drug toxicity and improve drug efficacy *in vitro* and *in vivo*.

## Introduction

Hepatocellular carcinoma (HCC) is one of the most universal tumour worldwide and accounts for 70–85% of liver malignancies. It has been ranked as the second leading cause of cancer-related mortality due to its poor progress (1, 2). The characteristic high mortality of HCC and deficiency of effective therapy are attributed to multi-factors, such as difficulty related to early diagnosis, high rates of metastasis and recurrence (3). Therefore, it is urgent to find more effective therapies.

Oxaliplatin, a third-generation platinum compound with the 1,2-diaminocyclohexane carrier ligand has become an important component of the front-line combination chemotherapy for colon cancer (4). Until now, many clinical therapies have been demonstrated a significant curative effect in digestive system neoplasms such as colorectal cancer, gastric cancer, and liver cancer (5–7). Oxaliplatin exerts its cytotoxic activity by forming DNA cross-links to inhibit nucleic acid polymerases and induce cell apoptosis. However, like other chemotherapeutic agents, oxaliplatin usually causes peripheral neuropathy, diarrhoea, and low blood cell counts (8–10). Those side effects cannot be endured by patients with oxaliplatin treatment, limiting the potential therapeutic effects of their clinical application (11).

The tyrosine kinases c-Met (mesenchymal-epithelial transition factor) is a transmembrane receptor for hepatocyte growth factor (HGF) of ligands and is expressed on the surfaces of various epithelial cells during embryogenesis to model tissue, which is controlled by the tumour suppressor p53 (12–14). c-Met plays a critical role in the development of malignancies such as liver cancer, non-small cell lung cancer, and melanoma (15–18). Amplification or mutation of the proto-oncogene MET can upregulate overexpression of c-Met, and it activates multiple downstream signalling pathways such as PI3K/AKT and RAS/ERK (19, 20). c-Met activation leads to multiple cellular biological processes such as proliferation, invasion and epithelial-mesenchymal transition (21). In HCC, ~50% of patients present c-Met aberrations (22). Moreover, c-Met overexpression is related to poor prognosis in patients. Due to an extremely important role of the abnormal function in the progress of HCC, c-Met has been identified as a therapeutic target. Previous studies showed that inhibitors of c-Met can decrease proliferation and invasion against c-Met-positive tumours (23, 24). Sorafenib, a monoclonal antibody targeting c-Met, has been approved for the treatment of advanced HCC by the Food and Drug Administration (FDA). Two randomized, placebo-controlled Phase III trials (Sorafenib Hepatocellular Carcinoma Assessment Randomized Protocol (SHARP) trial and the Asia-Pacific trial) demonstrated a significant improvement in overall survival (OS) in patients with advanced HCC (25, 26). However, Budhathoki and Shah utilized the SEER database to analyse the survival of HCC and showed a continued poor prognosis after the approval of sorafenib (27). Moreover, Fu's data showed that sorafenib caused hypertension in 38 patients (33.3%) and diarrhoea in 28 patients (24.4%) (28). Based on the comprehensive study, the development of more effective therapeutic strategies is urgently needed in the treatment of HCC.

Antibody-drug conjugates (ADCs) are a novel molecular targeted therapy in cancer treatment which have been demonstrated a promising antineoplastic drug with a wider therapeutic index (TI) than conventional chemotherapeutic agents (29). This new combination of medicines consists of the specific antibody, the therapeutic agent, and a valid linker. The aim of designing these drugs is to reach the specific target antigen of the tumour and deliver the therapeutic cytotoxic substance into cells while sparing the normal tissues (30). Until now, three ADCs have been approved by FDA for the therapy of certain types of cancer: gemtuzumab ozogamicin (acute myeloid leukaemia), brentuximab vedotin (Hodgkin's lymphoma and large-cell lymphoma), and T-DM1 (HER2-positive breast cancer) (31–33). Two critical parameters of ADCs with therapeutic potential are selectivity and efficacy. A better understanding that avoids antigen-independent toxicities of ADCs in normal tissues and that provides stability and sufficient cytotoxic agent to the tumour site is an essential and significant element. Over the past few years, many types of research have already supported c-Met as a targeting agent for anticancer therapy (34, 35). A novel ADC (ABBV-399) has been reported to induce effective cytotoxic activity in the c-Met-positive tumour (36). Oxaliplatin is the first-line treatment for operable and locally advanced tumours. A large number of nanodelivery techniques for platinum or platinum combined with monoclonal antibodies have been designed by many researchers (37, 38). However, there was no report referred to the utilization of oxaliplatin in the ADC design. Thus, we chose humanized c-Met IgG as a representative antibody and oxaliplatin as an administered cytotoxin in our study.

In this study, we describe novel therapeutic ADC strategies to conjugate oxaliplatin and humanized antibody (anti c-Met IgG) via a dipeptide-p-amidobenzyl alcohol linker as an active cancer-targeting agent. *In vitro* and *in vivo* experiments, we demonstrate an illustrative data set for balancing antitumour efficacy and high selectivity using a monoclonal antibody or chemotherapeutic drug treatment alone. Moreover, we also show that this ADC agent is highly effective in the treatment of c-Met-positive HCC.

## Materials and Methods

### Ethics Statement

This study was approved by the Ethical Committee of Nanjing Medical University. All the animal experiments were approved by the Animal Ethical and Welfare Committee of Nanjing Medical University, and carried out in accordance with recommendations of Animal protection, animal welfare and ethical principles, Institutional Animal Care and Use Committee (Approval No. IACUC-1703027).

### Cells and Agents

The HCC cell line HepG2 was obtained from the cell bank of Shanghai Institute of Biochemistry and Cell Biology. The HepG2 cell line was positive for c-Met expression (30–33). The cells were maintained in DMEM (Invitrogen, USA) supplemented with 10% (v/v) foetal bovine serum (Invitrogen, USA) and 1% (v/v) penicillin-streptomycin (Invitrogen, USA) in an atmosphere of 5% CO2 at 37°C. It was used within 3 months after resuscitation, and we did not repeat the cytogenetic testing. However, all the cell lines were monitored by our group for principal growth features (morphology and growth rate) and c-Met expression before use in experiments by the flow cytometry assay. *Escherichia coli* DH5 alpha was obtained from the Invitrogen company in the United States. The variable regions of anti-c-Met Fab, anti-TEX IgG, and 293 FreeStyle cells were preserved using the Key Laboratory of Antibody Technique of Ministry of Health of Nanjing Medical University (39).The IgG antibody eukaryotic expression vector pFUSE-CHIg-hG1, pFUSE CLIg-hκ, and 293F expression medium were acquired from Invitrogen company, USA. Oxaliplatin was produced by Shanghai YuanYe Biological Technology Company (Shanghai, China). Amicon tubes with membranes of 10,000, 30,000, and 50,000 MWCO were obtained from Millipore Corporation (Billerica, MA, USA).

### shRNA for c-Met in HepG2 Cells

c-Met shRNA (sense primer: 5′-GTCAAGCTTGAATTCCCCAGTGGAAAGACG-3′'; antisense primer: 5′-GTCGAATTCAAGCTTCCAAAAAAAATTAGTTCG-3′) were designed, synthesised and subcloned into the pSP72-E3 Ad shuttle vector (2).The plasmids were transfected into HEK-293T cells with Lipofectamine 3000 (Invitrogen, USA). Next, the lentiviruses in the supernatants were gathered and used to infect HepG2 cells. shRNA lentiviruses that mediated the silencing of c-Met were analysed by RT-PCR, qRT-PCR and Western blotting (Supplement 2).

### Western Blotting

Total cellular protein was extracted from shMet-HepG2 cells using RIPA solution according to the manufacturer's protocol. The cell lysate was electrophoresed through a 10% denaturing polyacrylamide gel and transferred onto a PVDF membrane (Bio-Rad, USA). The membrane was blocked with 5% non-fat milk and probed with the anti-c-Met antibody (Abcam, MA) at 4°C overnight. The blot was reacted with HRP-conjugated goat anti-rabbit IgG (Sigma-Aldrich, USA) at room temperature for 1 h, and the bands were detected with chemiluminescent substrate as suggested by the manufacturer (Bio-Rad, USA).

### Quantitative Real-Time PCR (qRT-PCR)

Total RNA of cells was extracted with TRIzol reagent (Invitrogen. USA), and cDNA was synthesised by reverse transcription with a Reverse Transcription Kit (Invitrogen. USA). The expression of related genes was quantified by qRT-PCR using SYBR Green (Takara), with GAPDH as a control. The primer sequences used for qRT-PCR were as follows: GAPDH (F) 5′-AGAAGGCTGGGGCTCATTTG-3′ and (R) 5′-AGGGGCCATCCACAGTCTTC-3′; c-Met (F) 5′-AATACGTGACGTAGAAAGTA-3′and (R) 5′-CATGGCTCTAGTTGTCGAC-3′. The fold change was calculated by the 2-ΔΔCt method.

### Production of Humanized Antibody IgG Against c-Met

The antibody eukaryotic expression vector pFUSE-CHIg-hG1, pFUSE-CLIg-hκ was cut using restriction enzymes Fsp I and Bmt I. With c-Met Fab as the template, which was previously constructed in our laboratory (40), the antibody heavy chain and light chain variable region sequences were amplificated by Infusion PCR. The antibody variable region gene was ligated into the eukaryotic expression plasmid using the Infusion PCR Kit. Subsequently, the recombinant plasmid pFUSE-CHIg-hG1-Met-2H, pFUSE-CLIg-hκ-Met-2κ was transformed into competent *E. coli* DH5 alpha. Using the bacterial colonies, the positive insert of recombinant plasmid was identified by PCR amplification through GenScript (Nanjing) Co. Ltd.

The recombinant plasmid pFUSE-CHIg-hG1-Met-2H/pFUSE-CLIg-hκ-Met-2κ was transfected into 293 FreeStyle cells. After 6 days, the cell culture supernatant was collected and purified using the protein purification system consisting of a Hitrap Protein A pre-loaded column. The conditions for the large-scale expression and purification of the human immunoglobulin G (IgG) format against c-Met were detected by SDS-PAGE.

### Immunoprecipitation Assay and Mass Spectrometry

After preparing the dynabeads, Protein A/G Magnetic Beads were mixed with 10 μg c-Met IgG diluted in 200 μl PBS with Tween-20. The samples were incubated with rotation for 10 min at room temperature, and the tube was placed on the magnet and the supernatant removed. After washing by gently pipetting 3 times, the Protein A/G Magnetic Beads-Ab complex was mixed with a sample containing the antigen and incubated with rotation for 10 min at room temperature. Upon including 25 μl 1 × SDS-page Loading Buffer with Protein A/G Magnetic Beads-Ab-Ag, the sample was complexed for 10 min at 95°C and the tube placed on the magnet. Finally, the supernatant/sample was loaded onto a gel for mass spectrometric analysis.

### Conjugation of the IgG Antibody Molecule to c-Met With Oxaliplatin

Oxaliplatin was reacted with N-(3-dimethylaminopropyl)-N'-ethylcarbodiimide hydrochloride (EDC) and N-hydroxysuccinimide (NHS) to develop the active compound. The active compound was gradually added to a solution of anti c-Met IgG in PBS (pH = 8.4) and interreacted for 20 h at 4°C. The compound mixture was purified using 10 kDa centrifugal ultrafilters to filter out unreacted active oxaliplatin. The conjugated compound was qualitatively investigated by HPLC (Waters, Milford, MA, USA) using a C18 (4.6 × 250 mm, 5 μm) with UV detection at 280 nm. The oxaliplatin concentrations were detected by flameless atomic absorption spectrometry, and a micro bicinchoninic acid (BCA) protein assay kit was used to measure the concentrations of anti c-Met IgG and unrelated IgG (41).

### ELISA

For ELISA, 50 μl/well of recombinant human c-Met/ HGFR Protein (Sino Biological #10692-H03H) at 1 μg/mL in PBS was used to coat 96-well plates for 2 h at room temperature, followed by blocking using 5% non-fat milk in PBS at room temperature overnight. The plates were washed 4 times with PBST and then incubated with c-Met-IgG, c-Met-IgG-OXA, and TEX-IgG for 2 h at room temperature, which were added at the same gradient effective-antibody concentrations from 0.156 to 1 μg/mL in quintuplicate wells. The plates were washed 4 times with PBST and then incubated with horseradish peroxidase-labelled anti-IgG secondary antibody in quintuplicate wells at room temperature for 1 h. The plates were washed 4 times with PBST, and 100 μL of TMB (Pierce, #34028) was added to each well and incubated for 20 min at room temperature. The reactions were stopped by addition of 2 M H2SO4, and the optical density (OD) was read at 450 nm.

### Flow Cytometry for Analysis of the Binding Capacity

Cells were collected by trypsin digestion to obtain single-cell suspension when the cells reached a confluence of 80% in 15 mL cell culture flasks. They were incubated for 60 min at room temperature with 1.0 μg of anti-c-Met IgG-OXA and fluorescein isothiocyanate (FITC)-conjugated isotype control antibody (Catalog: 11-4301) at 4°C, followed by FITC-conjugated anti-human antibody (Invitrogen Catalog: H10101C). Subsequently, the culture flasks were washed three times with PBS buffer to remove the unbound anti-c-Met reagent. The binding of the antibody to tumour cells was analysed using a BD Facscalibur with the Cellquest programme (Beckton Dickinson Biosciences, USA).

### Internalization Assay

Flow cytometry and cell immunofluorescence analyses were used to study the internalization kinetics of anti-c-Met IgG-OXA in HepG2 and shMet-HepG2 cell lines. The cells were seeded in 6-well plates and grown in DMEM with 10% FBS for 24 h. Then, 1 × 106 cells were washed with PBS, incubated with 10 μg anti-c-Met IgG-OXA and divided into 9 experimental groups and cultivated for 0, 5, 10, 15, 20, 40, 80, 120, and 180 min. The cellular supernatant was collected and placed on ice to stop the internalization. The flow cytometry assay was then performed using a FACSCalibur flow cytometer. Finally, the internalization percentage of different cells was calculated based on the mean fluorescence intensities (MFI) as follows: % Internalization =[(MFI Time ×-MFI background)/(MFI Time 0—MFI background)]×100.

A fluorescence microscope was used to observe the internalization effect directly. HepG2 and shMet-HepG2 cells were incubated with anti-c-Met IgG-OXA on ice for 20 min and then shifted at 37°C for 2 h. Then, the cells were incubated with a goat anti-human Alexa 488-conjugated secondary antibody (Thermo Fisher Scientific, USA) and 33342. Finally, the cells were washed with PBST and observed by fluorescence microscopy (Olympus, Tokyo, Japan).

### Cell Proliferation Assay

The Cell Counting Kit-8 (CCK-8) assay was used to analyse cell proliferation according to the manufacturer's instructions. HepG2 and shMet-HepG2 cells at a density of 5 × 105 cells/mL were seeded into a 96-well plate (100 μL/well). To evaluate the sensitivity to the drug, the cells were treated with oxaliplatin, anti-c-Met IgG, and anti-c-Met IgG-OXA at the same concentrations in different gradients. Cell proliferation was measured at 24 h. After treatment with the different drugs, CCK-8 reagent (Dojindo; Kumamoto, Japan) was then diluted in normal culture medium and added to each well. The cells were then incubated at 37°C and 5% CO_2_ for 4 h. When visual colour conversion occurred, absorbance at 450 nm was measured in each well with a microplate reader (Bio-Rad, Berkeley, CA, USA), and the percentage of cell proliferation was calculated.

### Wound Healing (Scratch) Assay

HepG2 and shMet-HepG2 cells were seeded in 6-well plates at a density of and cultivated at 37°C and 5% CO_2_ until they reached to 80–90% confluence. Subsequently, a 200 μl pipette tip was used to scratch a straight line in the cell monolayer. The cell debris was removed with PBS, and the cell culture medium was replaced with serum-free medium before every observation. Oxaliplatin, anti-c-Met IgG-OXA, and anti-c-Met IgG were added to each well at a concentration of 10 μM. Cells treated with 5% glucose solution served as a control. Images of cell movement into the wound area were photographed at 0, 24, and 48 h after initiation of the test using a digital camera system (Olympus, Tokyo, Japan). The cell migration distance (mm) was used to calculate the rate of migration in response to the different treatments.

### Cell Invasion Assay

Approximately 5 × 106 cells per well of HepG2 and shMet-HepG2 cell were seeded in 6-well plates and cultured in DMEM supplemented with 10% foetal bovine serum. When the cells reached 90% confluence, the cell culture medium was replaced with serum-free medium and incubated for 24 h. For the invasion assay, transwell plates (8 μm pore size, Corning, USA) pre-coated with Matrigel (Corning, USA) were used, and 100 μl suspension (2 × 105 starved cells) was seeded in the upper chamber in serum-free medium. As chemo-inducers, medium containing 10% FBS was added to the lower chamber. After incubation for 24 h, cells that remained above the Matrigel layer were removed using a cotton swab, and the migrated cells were fixed with methanol and stained with 0.1% crystal violet for 15 min. Images were obtained, and the cell quantity was counted in random microscopic fields in the insert.

### Flow Cytometric Analysis for Apoptosis Detection and Cell Cycle Analysis

After treatment with a concentration of 10 μM oxaliplatin, anti-c-Met IgG, and anti-c-Met IgG-OXA for 24 h, 1 × 106 cells were collected and used for apoptosis detection and cell cycle analysis. For cell apoptosis analysis, the cells were stained with the Annexin V-PE apoptosis detection kit (Fcmacs Co. Ltd.) according to the manufacturer's instructions. For cell cycle analysis, the cells were washed twice with PBS and fixed with 70% ethanol at 4°C for 14 h. The cells were incubated in staining solution for 30 min at room temperature and evaluated by flow cytometry with a FACSCalibur (BD Biosciences, USA). All the flow cytometry data were analysed using FlowJo software.

### Antibody-Dependent Cell-Mediated Cytotoxicity (ADCC) Assay

The LDH (lactate dehydrogenase) Cytotoxicity Assay Kit (Beyotime, Shanghai, China) was used to detect the capacity of PBMCs (effector cells) to trigger lysis of tumour cells in response to different drugs. Human peripheral blood mononuclear cells (PBMCs) were obtained from leukocytes, which were isolated from the peripheral blood of healthy donors. According to the guidelines of the Medical Ethical Committee of NJMU, all the donors in this study signed an informed consent. HepG2 and shMet-HepG2 cells (target cells) were seeded in 96-well plates (8 × 103 cells/well) overnight. For the ADCC assay, tumour cells (target cells) were incubated with a concentration of 10 μM anti-c-Met IgG and anti-c-Met IgG-OXA. PBMCs were incubated with tumour cells at an effector to target (E: T) ratio of 5:1, 30:1 and 100:1. After 4 h of co-incubation at 37°C, the cell supernatants were transferred to a 96-well plate, and LDH release was detected using the LDH Cytotoxicity Assay Kit (Beyotime, Shanghai, China) and quantified by measuring the absorbance at 490 nm according to the manufacturer's protocol. The cytotoxicity percentage of anti-c-Met IgG or anti-c-Met IgG-OXA was calculated as cytotoxicity% = 100 ×([experimental –effector spontaneous –target spontaneous]/[target maximum—target spontaneous]).

### Mouse Xenograft Tumour Model and Therapeutic Treatment *in vivo*

The *in vivo* anti-tumour activity of anti-c-Met IgG-OXA was monitored in nude mice bearing HepG2-luc (luciferase) cell hepatomas. Male BALB/c nude mice at 4 weeks of age were purchased from the Beijing Vital River Laboratory Animal Technology Co. Ltd. HepG2-luc cells in the logarithmic growth phase were subcutaneously injected at a total of 5 × 106 cells into the right armpit of each mouse. The tumour volume (TV) was calculated with calipers by the following formula: TV = (width)2 × length/2. When the tumours had reached 100–200 mm^3^ after inoculation, the mice were randomly assigned to six groups (6 mice per group): those treated with oxaliplatin (1 mg/kg and 10 mg/kg), anti-c-Met IgG-OXA (1 mg/kg, 5 mg/kg, and 10 mg/kg), and vehicle 5% sterile glucose solution. Different drugs were injected intravenously, and body weights were determined again in 3 days using bathroom scales. The mice were sacrificed when the tumours reached 1,500 mm^3^ or died due to cachexia. Tumours were rapidly excised, and the volume and weight were measured. After fixation in a paraffin block, immunohistochemistry (IHC) was used to observe the level of tumour cell proliferation. Additionally, the heart, lung, kidney, and spleen were excised and fixed to generate cross-sections for haematoxylin and eosin (H&E) staining. The protocol was approved by the Animal Ethical and Welfare Committee of Nanjing Medical University.

### Bioluminescence Imaging (BLI) *in vivo*

Six mouse groups were treated with different drugs, and the tumour status was monitored by BLI. Following injection with D-Luciferin potassium salt (Catalog: B24378-1 g), mice were anaesthetized with isoflurane. After 15 min, the nude mice bearing HepG2-luc cells were imaged for LUC signal by enterocoelic injection of 3 mg of D-Luciferin potassium salt in 5% glucose solution at 0, 7, 21, and 28 days after the different treatments. Animals injected with 5% glucose solution served as the untreated control. The BLI signals were analysed using a Xenogen IVIS 200 Imaging System.

### Adverse Drug Reactions in Mice

Mouse diarrhoea was considered an indicator of adverse reactions. The animals in each group were monitored three times daily, and the presence of diarrhoea was recorded. Drug-associated diarrhoea was graded based on the following parameters: 0, normal (no diarrhoea); (1) slight (staining of the anus); (2) moderate (staining over the thigh root); and (3) severe (staining over the abdominal region with continual oozing). At 2 days after the end of treatment, mice from all treatment groups were sacrificed, and the jejunum was collected and fixed in 10% buffered formalin and then assessed in haematoxylin and eosin (H&E)–stained cross-sections. After the mice were sacrificed, the spleens were removed, and the volumes and weights of the spleen were compared between the different treatment groups.

### Statistical Analysis

The data were statistically analysed with Prism v7.0 (GraphPad Software, Inc.). The data are presented as the mean ± SEM for the IC50 of the different treatments. The expression level of c-Met in the HepG2 and shMet-HepG2 cell lines was estimated by the two-tailed unpaired Student's *t*-test. The differences in therapeutic effect *in vitro* and *in vivo* after exposure to oxaliplatin and anti c-Met IgG-OXA were analysed by two-way ANOVA. The Student's *t*-test was adopted to compare two groups. The Kaplan-Meier method was used to analyse the OS data for the nude mice over 80 days. *P* < 0.05 was considered statistically significant in all comparisons.

## Results

### Production and Identification of Humanized Antibody

Identification of the recombinant chimeric plasmid pFUSE-CHIg-hG1-Met-2H, pFUSE-CLIg-hκ-Met-2κ was performed according to the size of the molecule by nucleic acid electrophoresis (Supplement 1A). As shows in the figure, the recombinant chimeric plasmids were composed of pFUSE-CHIg-hG1/ pFUSE-CLIg-hκ and anti-Met VH/Vκ Fab (324 bp/348 bp). *E. coli* efficiently amplified the recombinant chimeric plasmid, and the recombinant chimeric plasmids were transfected into 293F cells by 293 Fectin. Six days after cell transfection, the cellular supernatant was collected and purified by AKTA with a Protein A affinity column (Supplement 1B). The degree of purity of the samples produced was determined by SDS-PAGE (Supplement 1C). In the immunoprecipitation assay, anti-c-Met IgG was observed to combine with c-Met from the cell lysate (Supplement 1D).

### Preparation of Conjugates

**Figure 1:**
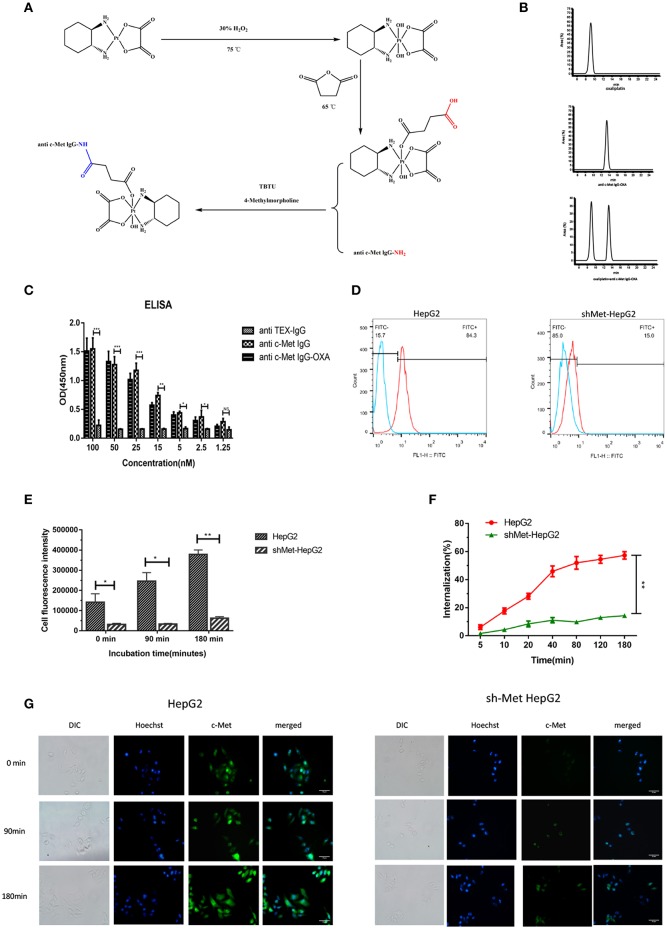
Synthetic schemes and characterization of humanized antibody IgG against c-Met with oxaliplatin. **(A)** A monoclonal antibody (anti c-Met IgG) connected by a non-cleavable linker to oxaliplatin using EDC/NHS chemistry. **(B)** RP-HPLC was used to identify anti-c-Met IgG conjugated to oxaliplatin at an absorbance wavelength of 280 nm. **(C,D)** The conjugate binding examination of the affinity of anti-c-Met IgG-OXA by the enzyme-linked immunosorbent assay, incubated with various concentrations of anti-c-Met IgG and anti-c-Met IgG-OXA, using anti-TEX IgG as the isotype control antibody. The signal-to-background ratios of anti-c-Met antibody revealed strong antigen-specific responses to c-Met protein. Moreover, the ELISA results also showed that the conjugates had the same affinity reactivity to the recombinant protein of c-Met. *p < 0.05, **p < 0.01, and ***p < 0.001. NS: not significant. Flow cytometry analysis of the binding of the HepG2 cell lines and shMet-HepG2 to anti-c-Met IgG-OXA was measured using FCM. Flow cytometry histograms demonstrating that anti-c-Met IgG-OXA bound specifically to the cell surface protein of c-Met. **(E–G)** The ratio of anti-c-Met IgG-OXA endocytosis was measured by flow cytometry for 0–180 min. The data showed that anti-c-Met IgG-OXA was internalized into HepG2 cells rapidly within 48 min and stabilized at 90 min. Compared with c-Met positive cell lines, there was a significant difference in shMet-HepG2 cells treated with the conjugates. The bars of internalization at 180 min represent the mean ± SD. Data represent three experiments performed in triplicate. *P < 0.05 and **P < 0.01. Different degrees of binding and internalization of the conjugates were observed in HepG2 and shMet-HepG2 cells by immunofluorescence microscopy (200×). Scar bars represent 10 μm. The degree of internalization of the conjugates in HepG2 cells increased over time, while the shMet-HepG2 cells showed barely any internalization.

By means of EDC/NHS chemistry, anti-c-Met IgG conjugation to oxaliplatin was coupled via the ε-amino groups of lysine (Figure 1A). The conjugates were analysed by RP-HPLC (Figure 1B). A trace of free oxaliplatin was detected at (tR) 9.2 min, and the peak for anti-c-Met IgG-OXA moved at (tR) 13.1 min. Almost no peak of free oxaliplatin was observed at 9.2 min in the anti-c-Met IgG-OXA group. These results indicated that anti-c-Met IgG and oxaliplatin were successfully conjugated. Moreover, using the BCA assay with BSA as a standard, the concentration of humanized antibody was detected, and the oxaliplatin concentrations were determined by flameless atomic absorption spectrometry. Finally, the average drug/antibody ratio of anti-c-Met IgG-OXA was 4.35, as shown in Table 1.

**Table 1:** Oxaliplatin /mAb ratio of anti-c-Met IgG-OXA.

**Concentration**	**Anti-c-Met IgG-OXA**
mAb concentration (mg/mL)^a^	2.56
Oxaliplatin concentration (mg/mL)^b^	15.1
Oxaliplatin /mAb molar ratio	4.35

a *The mAb concentration was measured using a BCA Protein Assay Kit*.

b *The oxaliplatin concentration was determined by flameless atomic absorptionspectroscopy (FAAS)*.

### Characteristics of Anti-c-Met IgG-OXA

To evaluate whether the drug/linker had changed the antibody affinity for c-Met antigen, ELISA, and flow cytometry were used to evaluate the binding affinity of anti-c-Met IgG-OXA to c-Met protein. As shown for the ELISA (Figure 1C), anti-c-Met IgG and anti-c-Met IgG-OXA specifically bound to c-Met protein in a similar dose-dependent manner. As expected, the unrelated antibody anti-TEX IgG hardly bound to c-Met, even at the highest concentration. Flow cytometry was utilized to test the selective cellular binding activity to anti-c-Met IgG-OXA (Figure 1D). The expression of c-Met in the shMet-HepG2 cell line clearly decreased based on the Western blot and qRT-PCR analyses. Images show (Figure 1D) the HepG2 and shMet-HepG2 cell line treated with anti-c-Met IgG-OXA, and the MFI value detected in HepG2 was much higher than in shMet-HepG2 cells (84.3% vs. 15.1%, P < 0.05), revealing that anti-c-Met IgG-OXA could specifically bind to c-Met-positive HepG2 cells. These results clearly suggested that conjugation of oxaliplatin to anti-c-Met IgG did not affect the idiosyncratic binding efficacy of the antibody after chemical conjugation.

### Internalization of Anti-c-Met IgG-OXA in Hepatocellular Carcinoma Cells

To determine whether anti-c-Met IgG conjugated to oxaliplatin was internalized upon binding to the cell surface associated with c-Met expression, the internalization rate of anti-c-Met IgG-OXA was quantified by flow cytometry and observed directly by fluorescence microscopy. In the flow cytometry assay (Figure 1F), anti-c-Met IgG-OXA was rapidly internalized into hepatic cells within 48 min. The internalization rate tended to stabilize at 90 min, and the rate of anti-c-Met IgG-OXA binding to HepG2 cells was 57.2%, exceeding that of anti-c-Met IgG-OXA to shMet-HepG2 cells (16.5%). As shown by fluorescence microscopy, the fluorescence intensity of HepG2 cells incubated with anti c-Met IgG-OXA was much higher than that of shMet cells (Figures 1E,G). These results are in accordance with the flow cytometry results and indicated that anti-c-Met IgG-OXA could be rapidly quickly and efficiently internalized into c-Met overexpressing cells.

### Cytotoxicity of ADC *in vitro*

**Figure 2:**
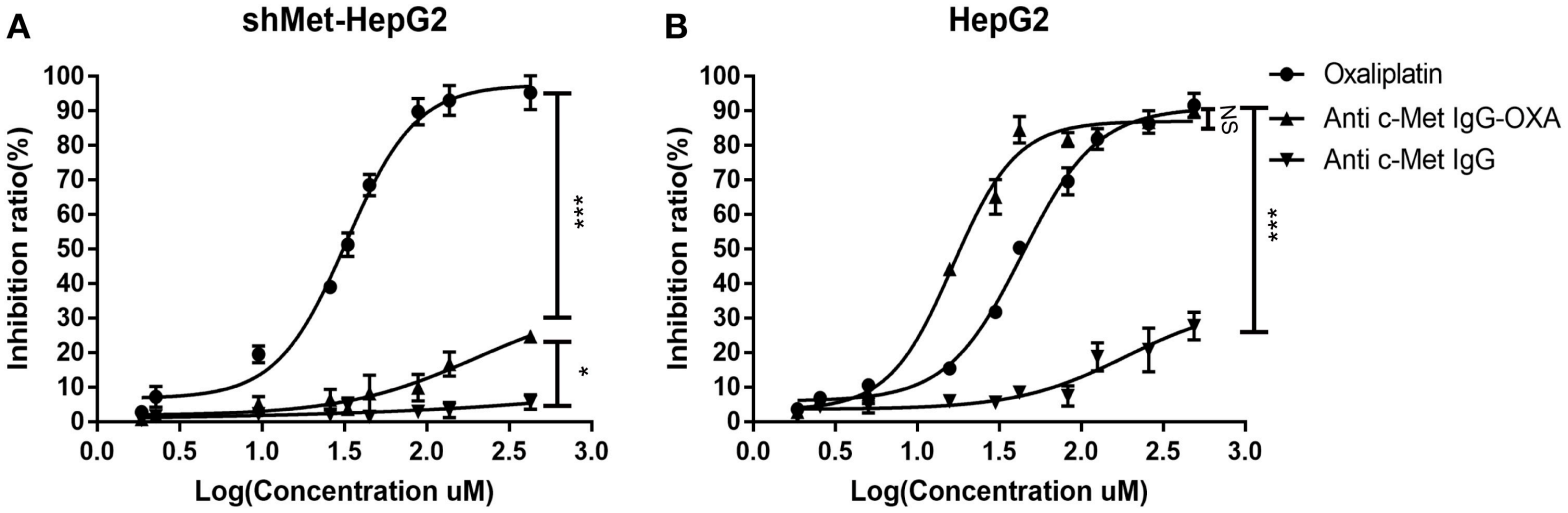
Proliferation of differentially c-Met-expressing cell lines detected using IC50 dose-response curves with the Cell Counting Kit-8 assay. **(A)** The proliferation of shMet-HepG2 cells decreased after treatment with oxaliplatin, while no significant effect was observed in the group exposed to humanized IgG or the conjugates after 24 h. **(B)** Oxaliplation and anti-c-Met-OXA inhibited the proliferation of the HepG2 cell line, and anti-c-Met IgG had no significant effect on cell proliferation. Statistical analysis was made of the inhibition ratio data at the maximum concentration among groups. Data are expressed as the logarithm change (average ± SD) from 5 random fields. NS, non-significant. *P < 0.05, ***P < 0.001 vs. “anti-c-Met IgG” as determined by ANOVA.

*In vitro*, the cytotoxicity of oxaliplatin, anti-c-Met IgG, and anti-c-Met IgG-OXA was evaluated using the HepG2 and shMet-HepG2 cell lines by the CCK-8 proliferation assay (Figures 2A,B). Using 5% isovolumetric glucose solution as a control, the calculated half maximal inhibitory concentrations (IC50) are listed in Table 2. As shown, oxaliplatin demonstrated obvious cytotoxicity in both cell lines based on the IC50 values. According to the concentration of effective-oxaliplatin, the cytotoxicity effects of ADCs and oxaliplatin on c-Met positive HepG2 cells were obvious with an IC50 value of 10.34 and 44.96 μM, which was in line with the previously budgeted drug antibody ratio of 4.35. This indicated that there was no change in the cytotoxic effect of oxaliplatin conjugated antibody molecules *in vitro*. However, it should be noted that anti-c-Met IgG-OXA showed a higher cytotoxicity to HepG2 cells with an IC50 value of 10.34 μM, whereas anti-c-Met IgG-OXA was much less cytotoxic to shMet-HepG2 cells (IC50 > 1,000 μM) under the same conditions. These results suggested that the cytotoxicity of anti-c-Met IgG-OXA was specific to the c-Met positive cell line HepG2 and was markedly decreased against the c-Met low expressing cell line shMet-HepG2.

**Table 2:** Cytotoxicity of oxaliplatin, mAb (anti-c-Met IgG), and ADC (anti-c-Met IgG-OXA) to the HepG2 and shMet-HepG2 cell lines.

**IC50 (μmol)**	**Oxaliplatin**	**Anti-c-Met IgG**	**Anti-c-Met IgG-OXA**
HepG2	44.96 ± 1.05	>1,000	10.34 ± 0.84
ShMet-HepG2	33.10 ± 1.92	>1,000	>1,000

### Impact on Invasion and Migration of ADC *in vitro*

**Figure 3:**
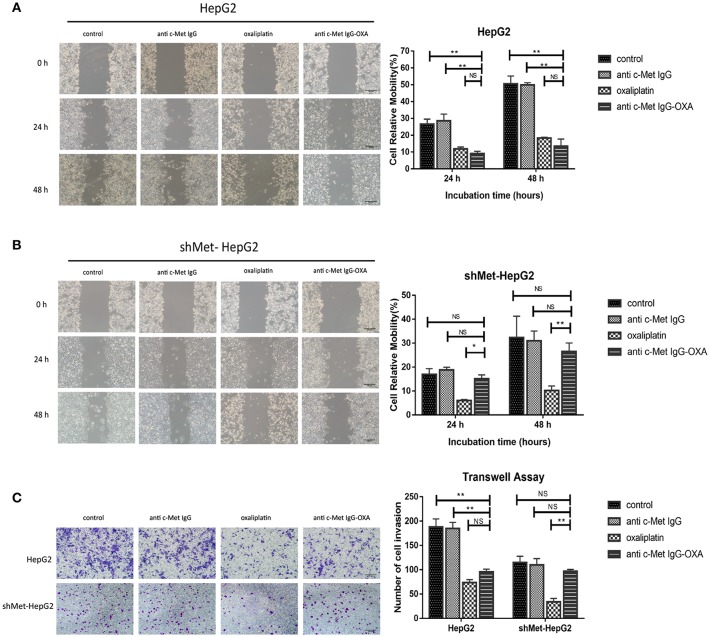
Scratch assays and transwell invasion assay. Scratch assays were performed to examine the migration capacity of two hepatocellular carcinoma cell lines after different treatments at 12, 24, and 48 h after wounding compared with 0 h. The transwell migration assay assessed the influence of the different treatments by counting the cell number that invaded through the transwell insert at 24 h. **(A,B)** At each time point, the scratch distance of HepG2 cells was significantly larger in the oxaliplatin and conjugate groups than in the control or humanized IgG group. In contrast, the effects of the conjugates on the migration of the c-Met knockdown cell line (shMet-HepG2) showed a significant higher healing rate compared with the oxaliplatin group. Scar bars represent 20 μm. **(C)** After treatment with oxaliplatin, anti-c-Met IgG-OXA, or anti-c-Met IgG, the transwell invasion assay with Matrigel was performed using HepG2 and shMet-HepG2 cells. The results showed that HepG2 cells treated with oxaliplatin or the conjugates invaded less than those treated with the humanized antibody or 5% glucose solution. The average number of transmembrane shMet-HepG2 cell in the conjugate was superior to the transmembrane cell number following treatment with oxaliplatin. The analysis is shown in the right panel. Images were acquired using an inverted microscope, and the quantitative data were analysed using the Kruskal–Wallis test. Data represent three experiments performed in triplicate. Scar bars represent 20 μm. *p < 0.05 and **p < 0.01.

We measured the migration and invasion of HepG2 cells and shMet-HepG2 cells after treatment by the wound healing assay and transwell assay. For the wound healing assay, the scratch distance was measured at 0, 24, and 48 h. The migration values showed that, compared with the untreated group, exposure to 10 μM oxaliplatin for 48 h significantly inhibited the closure of the scratched area in the HepG2 cell line (64.32%) (Figure 3A) and shMet-HepG2 cell line (68.28%) (*P* < 0.05 Figure 3B). HepG2 cells treated with anti-c-Met IgG-OXA showed a 53% scratch reduction. However, anti-c-Met IgG-OXA had no impact on the motility of shMet-HepG2 cells compared with the control group (*P* > 0.05).

To further explore the role of anti-c-Met IgG-OXA in tumour metastasis in HepG2 and shMet-HepG2 cells, transwell invasion assays were performed. Cells with different treatments and starvation statuses were placed in the upper chamber of transwells and allowed to invade through the Matrigel using 10% FBS as an attractant. The data showed that oxaliplatin and anti-c-Met IgG-OXA both markedly decreased the HepG2 cell invasion rate (*P* < 0.001, Figure 3C). However, anti-c-Met IgG-OXA did not impact the invasion rate compared with oxaliplatin in shMet-HepG2 cells (*P* < 0.001, Figure 3C). These results suggested that anti-c-Met IgG-OXA had an inhibitory effect on migration and invasion in HCC cells depending on the expression levels of c-Met.

### The Effect of Cell Apoptosis and Cell Cycle Induced by Anti-c-Met IgG-OXA in Hepatocellular Carcinoma Cell Lines

**Figure 4:**
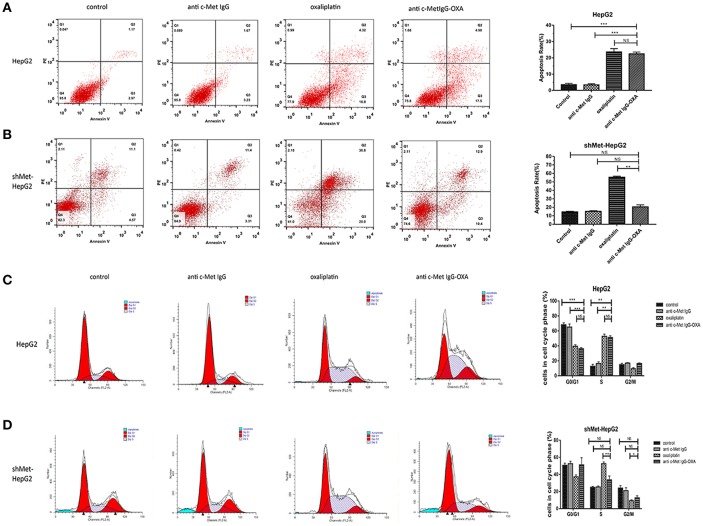
FCM cell apoptosis rate and cell cycle. The cell apoptosis rate and cell cycle were tested with the FCM assay during the course of treatment for 24 h. **(A,B)** Apoptosis of HepG2 and shMet-HepG2 cells induced by oxaliplatin, anti-c-Met IgG-OXA, anti-c-Met IgG, and 5% glucose solution was examined by FCM. Oxaliplatin induced apoptosis of HCC cells with or without c-Met knockdown. The anti-c-Met IgG-OXA had a significantly greater effect on the apoptosis of HepG2 than shMet-HepG2 cells. The apoptosis rate in response to anti-c-Met IgG and 5% glucose solution showed no significant effect in the two cell lines. **(C,D)** The various treatments of HepG2 and shMet-HepG2 cell line at each phase of cell cycle are shown in the cell cycle analysis diagram. The results exhibited significant cell cycle arrest at S phase with oxaliplatin treatment in the two cell lines. Compared with the shMet-HepG2 cell line, anti-c-Met IgG-OXA resulted in greater HepG2 cell arrest in S phase. Moreover, the cell cycle distributions of shMet-HepG2 cells in response to anti-c-Met IgG-OXA revealed no significant variation, in contrast to the humanized antibody and the control group. The apoptotic cell ratio and cell cycle phase distribution analysis are shown in the right panel. Values are the average ± SD. Data represent three experiments performed in triplicate. *P < 0.05, **P < 0.01, and ***P < 0.001.

To further determine the anti-proliferative effect in terms of cell apoptosis and cell cycle alterations with the different treatments, flow cytometric analysis was performed with the HepG2 and shMet-HepG2 cells treated with drugs for 24 h. The FCM results are shown in Figures 4A,B. In the HepG2 cell line treated oxaliplatin or anti-c-Met IgG-OXA at the same concentration (*P* < 0.05), the cell apoptosis rate was higher compared with the cells treated with antibody or 5% glucose solution. However, anti-c-Met IgG-OXA showed no significant difference in apoptosis compared with the control group and antibody group in c-Met low expressing cells (shMet-HepG2) (*P* > 0.05).

After treatment for 24 h, oxaliplatin induced a greater proportion of cells in S phase compared with the control group in the two cell lines (Figures 4C,D). Anti-c-Met IgG-OXA led to S phase cycle arrest as observed for oxaliplatin in shMet-HepG2 cells, which had scarcely any impact on cell cycle alterations (*P* > 0.05). The apoptosis and cell cycle analysis in HepG2 and shMet-HepG2 cells showed that anti-c-Met IgG-OXA had significantly a stronger selective anti-proliferative effect in the absence of oxaliplatin.

### ADCC Mediated by Anti-c-Met IgG-OXA in Hepatocellular Carcinoma Cell Lines

To address whether conjugation to oxaliplatin altered the ability of humanized IgG antibody to induce antibody-dependent cellular cytotoxicity (ADCC), HepG2, and shMet-HepG2 cell line were tested for their sensitivity to PBMC-mediated cytotoxicity after exposure to PBMCs collected from several healthy donors, according to the LDH Cytotoxicity Assay Kit.

**Figure 5:**
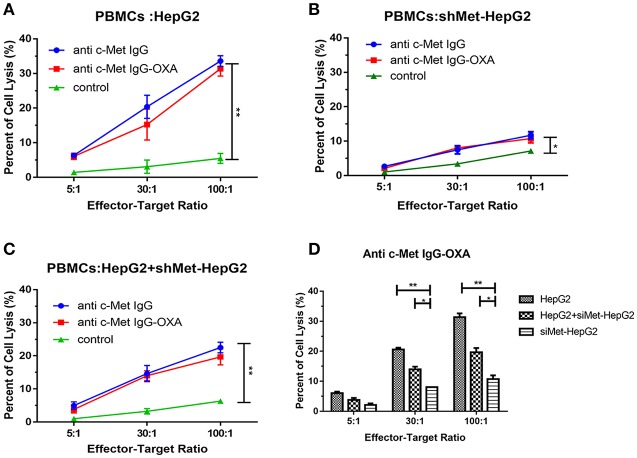
Antibody-dependent cell-mediated cytotoxicity. PBMC-mediated lysis of HepG2 and shMet-HepG2 cells was determined using a standard LDH Cytotoxicity Assay Kit in the presence of Fc fragment from the conjugates and humanized antibody. PBMCs as effector cells react with the HepG2 **(A)**, shMet-HepG2 **(B)**, or a mixture of HepG2+ shMet-HepG2 **(C)** target cells at a ratio of 5:1, 30:1, and 100:1 co-incubated with anti-c-Met IgG or anti-c-Met IgG-OXA. The humanized antibody and conjugates significantly induced ADCC in comparison to the control at all E:T ratios in HepG2 cells. A significant decrease in ADCC activity was observed in shMet-HepG2 cells with c-Met knockdown. Similar attenuation effects of ADCC could also be found in mixed HepG2+shMet-HepG2 cells. **(D)** Comparative analysis of the capacity of PBMC-mediated lysis in the presence of anti-c-Met IgG-OXA. No significant ADCC was stimulated in shMet-HepG2 cells. In the presence of the conjugate, PBMCs killed more HepG2 cells. Data represent three experiments performed in triplicate.*P < 0.05 and **P < 0.01.

The results showed that both humanized antibody and anti-c-Met IgG-OXA were similarly effective in inducing strong ADCC against the high c-Met-expressing cell line (HepG2) (Figure 5A). In the low c-Met-expressing cell line (shMet-HepG2), negligible ADCC was detected following incubation with PBMCs and anti-c-Met IgG-OXA or humanized antibody IgG compared with the control (Figure 5B). Next, we mixed HepG2 cells with shMet-HepG2 cells at a 1 to 1 ratio and exposed the mixed cells to PBMCs in the presence of anti-c-Met IgG-OXA or the humanized antibody IgG (Figure 5C). The results revealed moderate ADCC compared with the above groups (*P* < 0.05) (Figure 5D).

### ADC Enhanced Antitumour Activity in the HepG2-luc Xenograft Mice Model

To verify the present findings, we conducted an experiment by treatment of the high-dose oxaliplatin group (10 mg/kg), low-dose oxaliplatin group (1 mg/kg), three ADC dose groups (1 mg/kg, 5 mg/kg, 10 mg/kg concentration of oxaliplatin) and 5% glucose group. Compared with the 5% glucose treatment group, the high-dose oxaliplatin group, and three ADC dose groups showed obvious anti-tumour activity, while the low-dose oxaliplatin group showed no difference.

**Figure 6:**
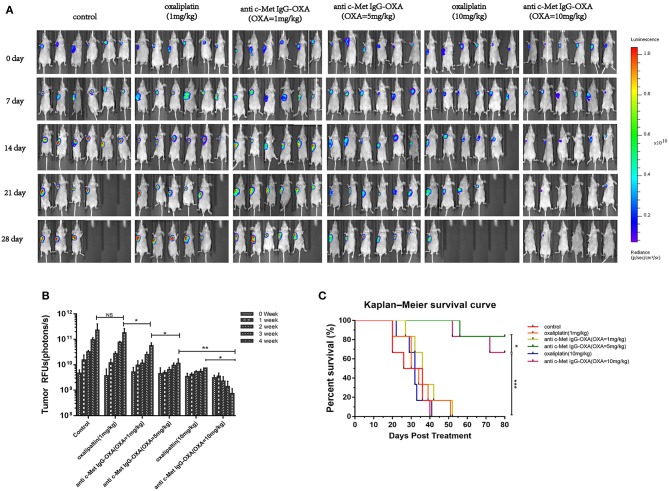
*In vivo* optical imaging analysis performed using the optical imaging system IVIS200 in mice bearing HepG2-luc cells. **(A)** Bioluminescence signal measured once a week after treatment with free oxaliplatin or anti-c-Met IgG-OXA in nude mice. The fluorescence image showed the lowest intensity NIRF signal for the tumour site in the conjugates than with the same dose of free oxaliplatin. **(B)** A quantitative graph of the tumour is depicted for each group with the signal-to-background ratio. **(C)** Kaplan–Meier survival analysis of nude mice (*n* = 6 for each condition) following tail-vein injection of free oxaliplatin or anti-c-Met IgG-OXA and monitoring over 80 days. A significant serious burden of adverse drug reactions was observed with the high dose of oxaliplatin (10 mg/kg). The percentage of remaining survival in the high-dose oxaliplatin group (10 mg/kg) (33.33%) was lower than that in the control group (50%) at 28 day, which was far below the group treated with ADC (10 mg/kg) (100%). ADC demonstrated a significant improvement of the survival rate compared with the same dose of oxaliplatin and the control group. *P < 0.05, **P < 0.01, and ***P < 0.001.

The animals were imaged post-injection of D-Luciferin potassium Salton using a Xenogen IVIS 200 optical imaging system for the first injected drugs when the tumour volume reached 100–200 mm^3^, which was marked as 0 days, and subsequently imaged four times at 4 weeks. The images showed that the optical bioluminescence imaging signal was determined by the number of tumour cells treated with different concentrations of drugs in nude mice bearing a HepG2-luc tumour (Figures 6A,B). Compared with the 5% glucose treatment group, the signal in the high-dose oxaliplatin group (10 mg/kg) was weaker and much smaller in the volume range (*P* < 0.05) over time, while there were obvious changes in the image signal and tumour volume following treatment with the dose of ADC (with the same concentration of 10 mg/kg oxaliplatin). Compared with the above treatment group, the low-dose oxaliplatin group (1 mg/kg) showed no difference from the 5% glucose treatment group, while the low-dose ADC (1 mg/kg) was obviously decreased (*P* < 0.05). Moreover, ADC substantially prolonged the overall survival of mice bearing a HepG2-luc tumour compared with the same dose of oxaliplatin and the control group (Figure 6C).

**Figure 7:**
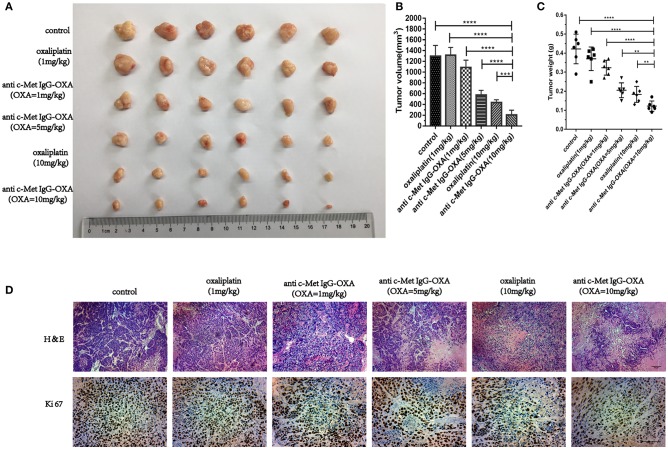
The humanized antibody IgG against c-Met enhances the *in vivo* efficacy of oxaliplatin. **(A–C)** Six BALB/c nude mice bearing subcutaneously implanted HepG2-luc tumours per group were injected i.v. with different concentrations of ADC or oxaliplatin as described above in the original text. Tumour volumes and weight were monitored following treatment at day 33. At the same concentration of oxaliplatin, anti-c-Met IgG-OXA resulted in significant tumour growth inhibition compared with free oxaliplatin. **(D)** Tumour samples were collected after sacrifice, and H&E (100×) and Ki67 (200×) were estimated by immunohistochemical analysis. Scar bars represent 20 μm. Data are represented as the mean ± SD, *n* = 6 for each condition. **P < 0.01, ***P < 0.005, and ****P < 0.001.

The tumours of nude mice were recording to evaluate the *in vivo* effects of various treatments when all mice were sacrificed (Figures 7A–C). The tumour inhibition ratios after various treatments were statistically calculated and showed that ADC (5 mg/kg) and oxaliplatin (10 mg/kg) had similar effects on tumour inhibition (*P* > 0.05), and there was a significant difference in the tumour volume between the ADC and oxaliplatin treatments with an equivalent oxaliplatin dosage of 1 mg/kg (*P* < 0.05). To further evaluate ADC inhibition of HepG2 cell proliferation in nude mice, we examined the tumour samples by H&E and Ki-67 staining (Figure 7D). The results demonstrated a significant decrease in Ki-67 staining in the tumour tissue sections after treatment with 5 mg/kg ADC or 10 mg/kg oxaliplatin, and the above phenomenon was even more evident following treatment with a high dose of ADC (10 mg/kg). Taken together, these results indicated that ADC showed more effective cancer-killing effects compared with the free oxaliplatin, which provided an equivalent effect.

### Anti-c-Met IgG-OXA Attenuates the Side Effects Induced by Oxaliplatin

**Figure 8:**
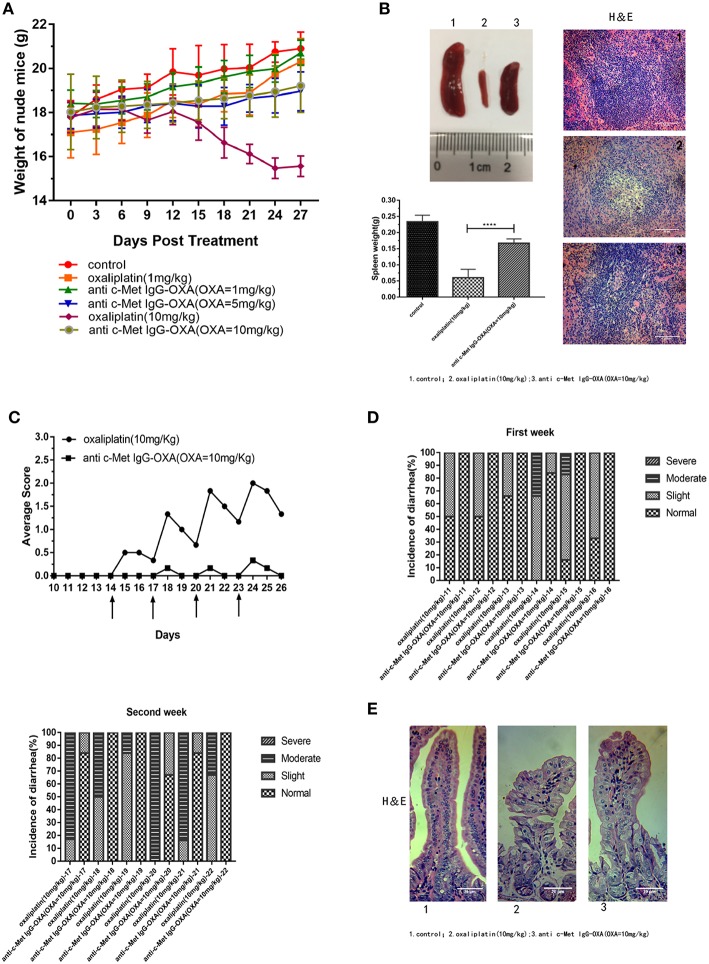
**(A)** The humanized antibody IgG against c-Met ameliorated cachexia, diarrhoea, and atrophy of the spleen in HepG2-luc-bearing nude mice treated with oxaliplatin. Average body weights were record over time, and the weights in the high-dose oxaliplatin-treated mice were significantly lower than in the other groups. The body weights of mice in the anti-c-Met IgG-OXA (10 mg/kg) group never declined significantly compared with the control group. **(B)** Representative images of spleens from the mice treated with free oxaliplatin (10 mg/kg), the conjugates (10 mg/kg), and 5% glucose solution. Moreover, histopathological examination of the spleen is shown in the right panel (H&E staining at 200x magnification), and the spleen weight is recorded in the left panel. Anti-c-Met IgG-OXA clearly prevented spleen atrophy compared with free oxaliplatin at an oxaliplatin dose of 10 mg/kg. **(C,D)** Mice were orally administered the oxaliplatin and anti-c-Met IgG-OXA with an equivalent oxaliplatin concentration (10 mg/kg) every 3 days when the tumour volume reached 100–200 mm^3^ at day 14. The experimental protocol for monitoring diarrhoea was followed as stated in the original text using the four-grade scale (0 to 3). By 14 days of age, diarrhoea average scores and incidence rate of diarrhoea in the mice were recorded daily. The severity and frequency of diarrhoea were significantly attenuated in the conjugate-treated mice, while severe diarrhoea was observed in the mice treated with free oxaliplatin. The arrows indicate treatment days. **(E)** Tissue sections of the small intestinal epithelium of the oxaliplatin- and anti c-Met IgG-OXA-treated mice were observed by haematoxylin-eosin staining (200x magnification). The villus length and structure of the gland in response to the different treatments showed highly disrupted gland tissues in the free oxaliplatin group (10 mg/kg). In contrast, the conjugates protected the small intestinal epithelium against serious damage. Data represent the mean ± SD, *n* = 6 for each condition. Scar bars represent 20 μm.

Compared with anti-c-Met IgG-OXA or high-concentration oxaliplatin treatment, we evaluated the incidence of diarrhoea, morphology of the intestine, and change in body weight to investigate whether anti-c-Met IgG-OXA could alleviate the side effects of oxaliplatin. After initiating the various treatments, the weights of nude mice were dynamically recorded every 3 days to evaluate the *in vivo* side effects of various treatments until all the mice were sacrificed. As shown in Figure 8A, mice treated with oxaliplatin (10 mg/kg) appeared to lose weight with an absence of orexis. Moreover, a great reduction of the splenic size was observed in the oxaliplatin treatment group compared with the anti-c-Met IgG-OXA treatment group (Figure 8B). Daily monitoring data of diarrhoea showed that the incidence and severity of diarrhoea were decreased in the anti-c-Met IgG-OXA treatment group, which was one of the most common side effects of oxaliplatin (Figures 8C,D). To further investigate the attenuation of diarrhoea by anti-c-Met IgG-OXA, the jejunum tissue was sectioned for histological examination. The pathological tissue showed that the structure of the jejunum mucosa showed better preservation in the anti-c-Met IgG-OXA group compared with the oxaliplatin group (Figure 8E).

**Figure 9:**
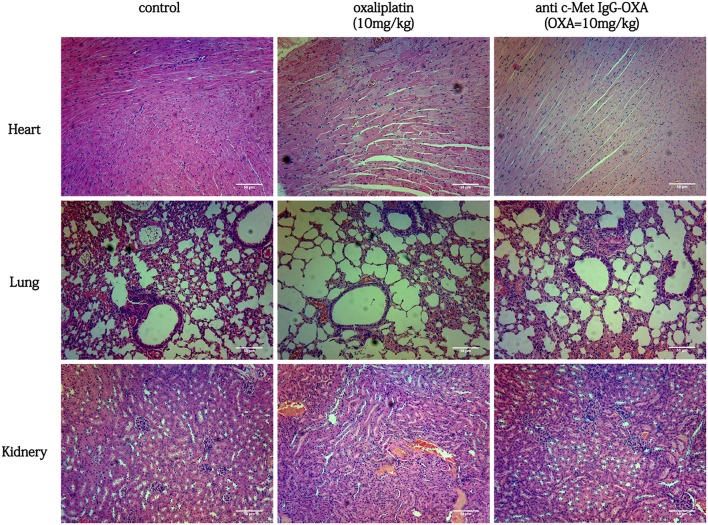
Haematoxylin and eosin staining of the heart, lung, kidney, and liver isolated from mice with various treatments (100x magnification). In the group of conjugates (10 mg/Kg), minimal tissue injury was observed in the organ sample compared with free oxaliplatin. In a comparison of the different treatments, anti-c-Met IgG-OXA showed greater advantages and fewer side effects *in vivo*. Scar bars represent 50 μm.

In the tissue pathology examination (Figure 9), the myocardial filament, lung alveolus, and kidney glomerulus were found to have different levels of injury in the oxaliplatin treatment group (10 mg/kg). However, barely any injury in the above organization was observed, even after high-dose treatment of anti-c-Met IgG-OXA. In the anti-c-Met IgG-OXA-treated mice, there was no obvious visible disruption of the myocardial filaments and vacuolization compared with the oxaliplatin -treated mice. Severe hyperaemia and haemorrhage in the lung alveoli were also discovered in the oxaliplatin-treated mice, a phenomenon secondary to heart failure. The extensive atrophy of the kidney glomerulus observed in the oxaliplatin-treated mice was quite serious, while slight changes were detected following treatment with anti-c-Met IgG-OXA. The above results suggested that anti-c-Met IgG-OXA could reduce the cytotoxicity to the non-target organs.

## Discussion

ADCs are encouraging as efficient tools for the targeted delivery of small-molecule drugs to cancer *in vitro* and *in vivo*, coupling the therapeutic effect of the cytotoxic drug to a highly specific mAb (42–44). To date, some research has demonstrated that ADCs exhibit promising anticancer therapy in malignancy. Four ADCs have been approved by the FDA: gemtuzumab ozogamicin, inotuzumab, ozogamicin, and brentuximab vedotin (45, 46).The remarkable antitumour effect of ADCs is mainly determined by the following three structures: monoclonal antibody, stable linker, and small molecule toxic drugs. ADCs enhance the specific killing of tumour cells by interacting with specific antigens on tumour cells, with effective internalization and lysosome transition for the release of the cytotoxic drug by lysosomal proteolysis. The selection of a target is critical when effective ADCs are established. For this process, the target antigen on the tumour cells must be highly expressed compared with normal tissues (47). c-Met, with a potential tumour-promoting role (48), is clearly overexpressed in excess of 25% in HCC compared with normal liver at the protein level (49, 50). Wang et al. has confirmed that c-Met is a successful targeting agent for cytotoxic agent delivery (36). Thus, we chose c-Met as a representative surface antigen in our study.

In our work, by amplifying the variable region of anti c-Met Fab using a phage display technique, anti-c-Met VH/Vκ was cloned into the pFUSE-CHIg-hG1 and pFUSE-CLIg-hκ expression vectors (40). The humanized IgG1 was prepared using eukaryotic cells and showed a specific binding capacity to c-Met by immunoprecipitation and mass spectrometry assays. Next, we designed a drug delivery strategy based on oxaliplatin as an active cancer-targeting c-Met antigen. The oxaliplatin/antibody ratio of this novel conjugate was calculated to be 4.35:1. The conjugates retained the same specific binding ability to targeted cancer cells compared with anti-c-Met IgG. Compared with the shMet-HepG2 cell line, the synthesised conjugate showed more specific cytotoxicity in c-Met positive HepG2 cells *in vitro*. Similar to oxaliplatin, anti-c-Met IgG-OXA promoted apoptosis and cell cycle arrest mainly in S phase, with barely any impact on the shMet-HepG2 cells. The PBMC cell-based ADCC assay was applied to probe the effect of the Fc of IgG1 from anti-c-Met IgG-OXA *in vitro*. Our observations suggested that anti c-Met IgG-OXA maintained the ADCC induced by Fc of IgG1 (51). The results further showed that anti-c-Met IgG-OXA had slight utility in ADCC to kill cancer cells in shMet-HepG2 or mixed cells. In addition, the cell immunofluorescence and internalization assay showed the internalization of anti-c-Met IgG-OXA tended to stabilize after cultivated for more than 180 min. It may indicate that there exists a mechanism involving access of Met into a recycling pathway after internalization (52). ADC has different fates after entering the cell. For example, ADC reaches lysosomes where its linker of the ADC is degraded leading to the release of the drug after it binds to its surface antigen and the complex is internalized. Then, the drug passes from the intracellular compartment to the cytosol and binds to its target (53).The specific mechanism how anti-c-Met IgG-OXA works after internalization needs to be further studied in the future.

*In vivo*, we established a nude mouse model of HCC and detected the overgrowth of HepG2-luc cells with the whole-body fluorescent imaging system. As expected, our research confirmed that anti-c-Met IgG-OXA resulted in a sensitization to oxaliplatin chemotherapy with tumour killing. ADCs aim to utilize the specificity of monoclonal antibodies (mAbs) to deliver cytotoxic agents selectively to antigen-expressing tumour cells and minimize the associated untoward effects of conventional cancer drug therapy (46). In this study, our data showed that the conjugate significantly reduced the incidence of serious side effects, such as diarrhoea, weight loss and spleen size (54), suggesting that anti-c-Met IgG-OXA could reduce the side effects induced by oxaliplatin. Taken together, these conclusions confirm that the anti-c-Met IgG conjugate oxaliplatin is a remarkable and potent specific delivery system for chemotherapeutics to the tumour site *in vitro* and *in vivo*.

Additionally, several limitations should be noted in our study. First, anti-c-Met-IgG-OXA could selectively inhibit HepG2 cells with high c-Met expression compared with c-Met-low-expressing shMet-HepG2 cells. However, the dose range of anti-c-Met-IgG-OXA was not clear for the treatment of HepG2 cells. Second, compared with orthodontic tumours, many defects persisted in the transplanted tumour model, such as the tumour micro-environment and vascular density. Anti-c-Met-IgG-OXA was better able to mimic the process of tumour inhibition in the treatment of the orthodontic tumour. Moreover, we did not allow sufficient time to clearly determine the presence of any resistance phenomena after the treatment with anti-c-Met IgG-OXA. The *in vivo* efficacy of trastuzumab-DM1 and Lewis Phillips (55) showed that HER2-amplified breast cancer started to resist the ADC treatment at day 70. Finally, we did not consider the presence of soluble c-Met proteins in the systemic circulation. Soluble c-Met may affect the concentration of ADC in the blood, reducing the amount of drug reaching to the tumour cells. Concurrently, ADC and soluble c-Met proteins in the blood and NK cells and other immune cells function together to cause serious ADCC, resulting in off-target immune damage (56). To overcome these shortcomings, we could remodel the structure of the anti-c-Met IgG-OXA to improve the specific immunotherapy for the tumour cells. For example, the dual targeting ability of ADCs is a greater focus in molecular targeted therapy. Sugahara showed that the use of 5D5 anti-c-Met antibody and iRGD peptide-mediated drug provided a stronger suppressive effect compared with 5D5 anti-c-Met antibody-mediated or iRGD peptide-mediated drug (57–59). These findings reminded us to build bispecific antibodies-drugs coupled to the development of ADCs to improve drug targeting and dramatically reduce side effects due to off-target effects.

Briefly, an effective c-Met-targeting ADC has been manufactured with fully humanized mAb coupling oxaliplatin. This ADC enhances the therapeutic specificity among normal and cancer cells. This means that it can reduce severe side effects and improve the therapeutic efficacy of oxaliplatin for the treatment of HCC with c-Met overexpression. This molecular-targeted strategy could be extended to the treatment of other c-Met positive cancers to reduce side effects and improve anticancer bioactivity with a lower drug concentration.

## Data Availability

All datasets generated for this study are included in the manuscript and/or the Supplementary Files.

## Ethics Statement

This study was carried out in accordance with recommendations of Animal protection, animal welfare and ethical principles, Institutional Animal Care and Use Committee. The protocol was approved by the Animal Ethical and Welfare Committee of Nanjing Medical University.

## Author Contributions

YM, MZ, JW, XH, XK, XZ, YC, LJ, and ZF: experiments, manuscript development, and writing. QT and ZL: concept, supervision, and writing.

### Conflict of Interest Statement

The authors declare that the research was conducted in the absence of any commercial or financial relationships that could be construed as a potential conflict of interest.
