# Supplementary material for: High-Affinity Human Anti-c-Met IgG Conjugated to Oxaliplatin as Targeted Chemotherapy for Hepatocellular Carcinoma
Source: Front Oncol. 2019 Aug 2;9:717. doi: 10.3389/fonc.2019.00717 (PMC6688309; doi:10.3389/fonc.2019.00717)

**Supplementary Material**

**Supplement 1**

(A)Recombinant chimeric plasmid pFUSE-CHIg-hG1-Met-2H、pFUSE-CLIg-hκ-Met-2κ was identified by nucleic acid electrophoresis.(B)Humanized anti-c-Met antibody IgG was fully assembled from Freestyle 293 suspension cell and eluate was affinity purified from cell supernatant by AKTA purifier 100 with HiTrap protein A.UV curve of purification process was drawn by Unicorn 5.11 system.(C) Purified product was examined by non-reducing SDS-PAGE with Coomassie blue staining.Lane 1: original cell supernatant protein ladder; Lane 2: transfection cell supernatant; Lane;3:heavy and light chain of anti c-Met IgG; Lane 4: isotype control antibody IgG.(D)Purification of c-Met-specific humanized IgG was detected by immunoprecipitation and mass spectrometry analysis. Antibody pulldown in immunoprecipitates assay followed by Western blotting for detceting the antibody IgG specific for c-Met . Input(Total extract from HepG2 or shMet-HepG2 cells) and Eluted (elution extracted by antibody coupled with protein A/G magnetic bead from input) were shown by Western Blotting incubated with commercial Met antibody.The image for HepG2 and shMet-HepG2 cell lysate lanes exhibited the presence of c-Met at varying degrees .

**Supplement 2**

Validation of HepG2 cell line transfected with shRNA against c-Met was determined by qRT-PCR and immunoblotting,with GAPDH as loading control.As the data showed that c-Met knockdown decreased the expression of c-Met mRNA and protein in HepG2 cells.Data was presented as Mean ±SD n = 6.* P< 0.05.

**Supplement 1**


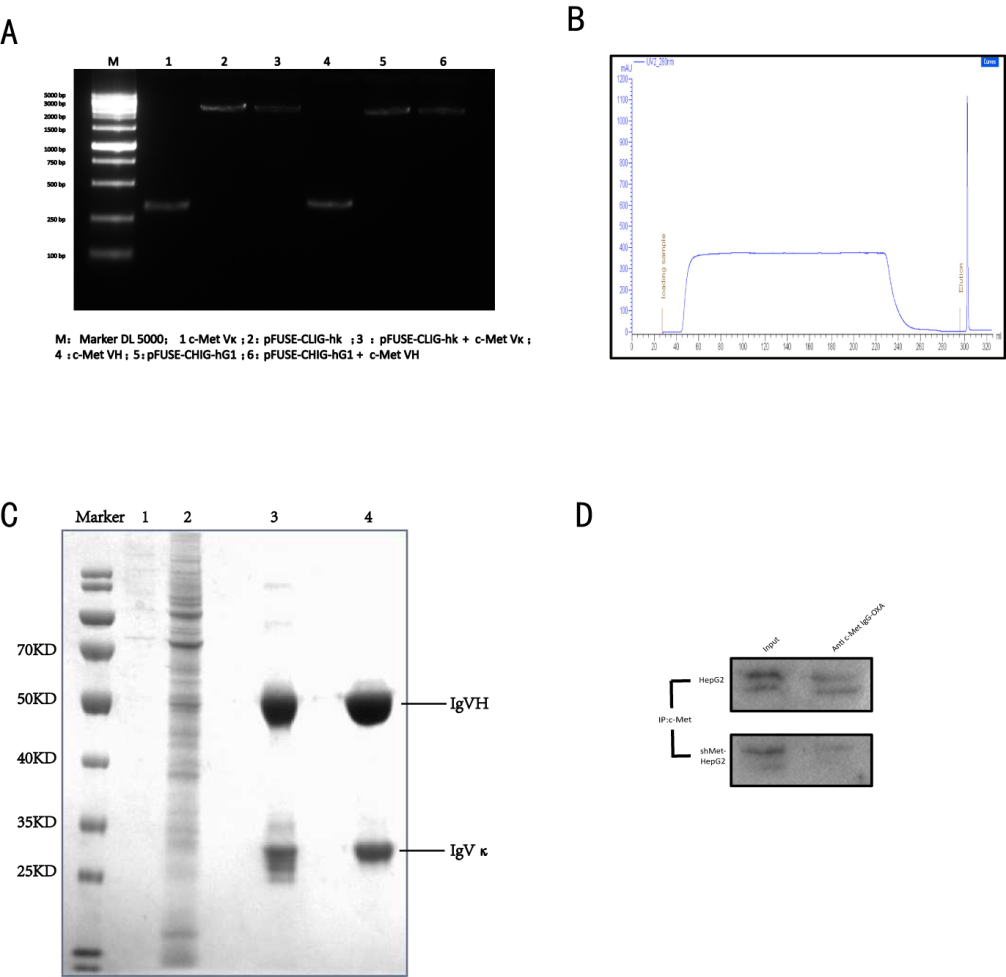


**Supplement 2**


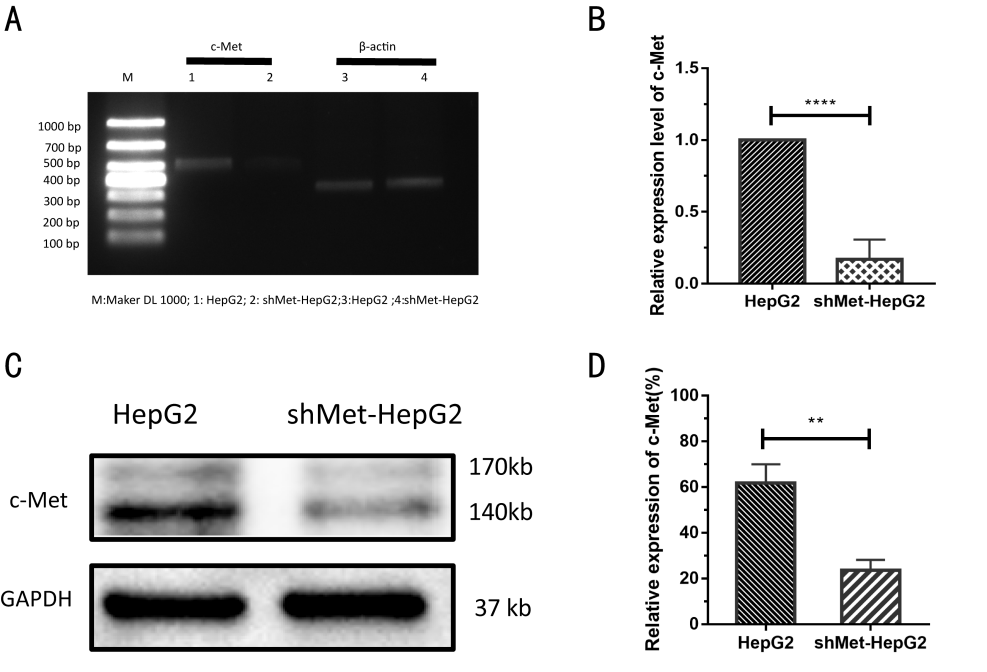

Supplement: Supplementary file 1 [file Data_Sheet_1.docx]
